# P-999. Differences in Epidemiology and Outcomes Between Toxin-Positive and Toxin-Negative Clostridioides difficile Infections

**DOI:** 10.1093/ofid/ofaf695.1197

**Published:** 2026-01-11

**Authors:** Amy Pulikeyil, Ali Ibrahim, Nicholas J Dante, Keith S Kaye, John P Mills

**Affiliations:** Rutgers Robert Wood Johnson Medical School, Franklin Park, NJ; Rutgers - New Brunswick, Edison, New Jersey; Rutgers Robert Wood Johnson Medical School, Franklin Park, NJ; Rutgers Robert Wood Johnson Medical School, Franklin Park, NJ; Rutgers Robert Wood Johnson Medical School, Franklin Park, NJ

## Abstract

**Background:**

Epidemiological characteristics and clinical outcomes of patients with PCR positive/toxin EIA-negative *C. difficile* infection (CDI) are poorly defined and treatment decisions in this subgroup often vary widely. Here, we assess differences in epidemiology and outcomes between toxin-positive and toxin-negative CDI.

**Figure:**
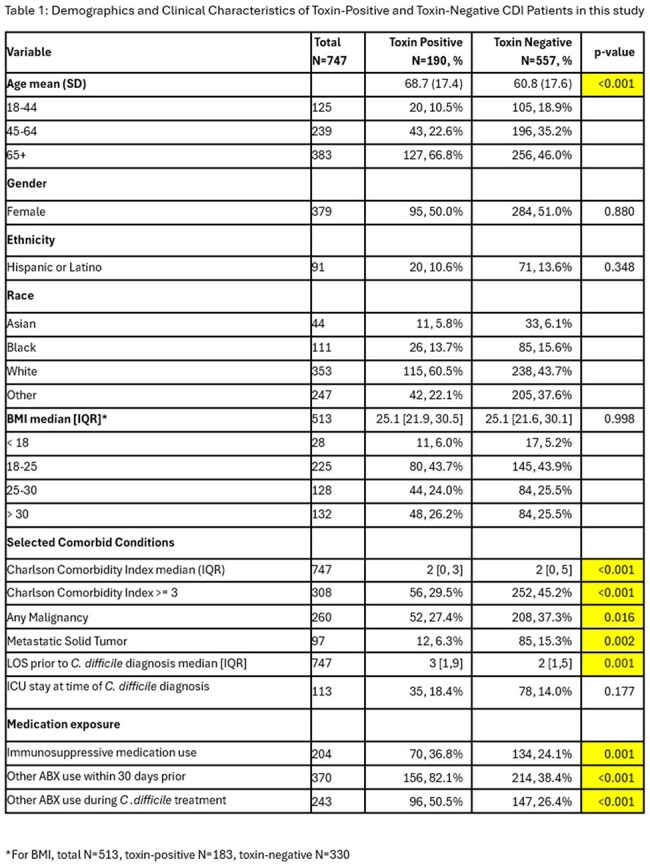

**Figure:**
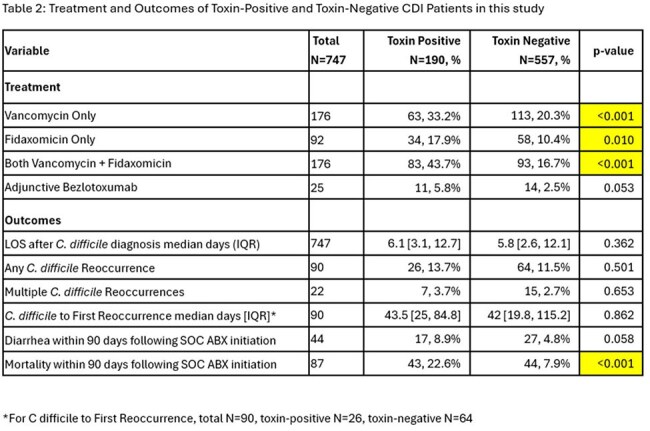

**Methods:**

We performed a retrospective analysis of adult patients with a positive *Clostridioides difficile* PCR test at RWJUH from 1/1/2018 to 12/31/2024. We assessed differences in epidemiology and outcomes between patients with toxin-positive and toxin-negative CDI.

**Results:**

We identified 747 patients with positive *C. difficile* PCR; 190 were toxin EIA-positive and 557 were toxin EIA-negative (Table 1). No significant differences were noted between the groups regarding gender, ethnicity or BMI. Toxin-positive patients were older compared to toxin-negative patients (Table 1). Comorbid conditions were similar between groups except for malignancies which were more common in toxin-negative CDI. The toxin-positive group had a longer length of stay prior to CDI and more frequent high-risk medication exposure (immunosuppressive medications, antibiotics). As expected, toxin-positive patients more frequently received anti-CDI therapy compared to toxin-negative group (Table 2). CDI recurrences and diarrhea within 90 days of treatment were more common in toxin-positive group but this did not reach statistical significance. Notably, toxin-positive group had a significantly higher incidence of mortality than the toxin-negative group (OR 3.41, 95% CI 2.15-5.40). In multivariate analysis, after controlling for differences between the two groups, toxin positivity remained significantly associated with mortality (OR 2.49, 95% CI 1.38-4.55).

**Conclusion:**

Patients with toxin-positive CDI were older and had a longer length of hospital stay with increased exposure to immunosuppressive medications and antibiotics. CDI recurrences and diarrhea within 90 days post treatment were more common in the toxin-positive group, but these differences were not statistically significant. Patients with toxin-positive CDI had a significantly higher mortality rate compared to those with toxin-negative CDI, despite the latter group having more chronic comorbid illness.

**Disclosures:**

Keith S. Kaye, MD, MPH, AbbVie: Advisor/Consultant|GSK: Advisor/Consultant|Merck: Advisor/Consultant|Shionogi: Advisor/Consultant

